# Three-dimensional regulation of transcription

**DOI:** 10.1007/s13238-015-0135-7

**Published:** 2015-02-12

**Authors:** Jun Cao, Zhengyu Luo, Qingyu Cheng, Qianlan Xu, Yan Zhang, Fei Wang, Yan Wu, Xiaoyuan Song

**Affiliations:** CAS Key Laboratory of Brain Function and Disease and School of Life Sciences, University of Science and Technology of China, Hefei, 230027 China

**Keywords:** transcriptional regulation, long non-coding RNAs, three-dimensional chromosome interactions, epigenetic changes

## Abstract

Cells can adapt to environment and development by reconstructing their transcriptional networks to regulate diverse cellular processes without altering the underlying DNA sequences. These alterations, namely epigenetic changes, occur during cell division, differentiation and cell death. Numerous evidences demonstrate that epigenetic changes are governed by various types of determinants, including DNA methylation patterns, histone posttranslational modification signatures, histone variants, chromatin remodeling, and recently discovered chromosome conformation characteristics and non-coding RNAs (ncRNAs). Here, we highlight recent efforts on how the two latter epigenetic factors participate in the sophisticated transcriptional process and describe emerging techniques which permit us to uncover and gain insights into the fascinating genomic regulation.

## TRADITIONAL MODEL OF TRANSCRIPTIONAL REGULATION IN EUKARYOTES

Phenotypic diversity of cells and their response and adaptation to the environment are achieved through the regulation of distinct gene expression in particular temporal-spatial context. In this context, transcription control is a crucial step (Levine and Tjian, 2003; Reik, 2007; Hager et al., 2009; Tsompana and Buck, 2014). Many studies indicate that aberrant transcriptional regulation is closely related with development and exacerbation of diseases (Newman and Young, 2010; Harismendy et al., 2011; Li et al., 2013a; Luft, 2014; Yang et al., 2014). Understanding how genome is orderly transcribed is vital for deciphering the mystery of cellular phenotypical changes and homeostasis.

In traditional studies, we usually think of genomes, which encode genetic information in their linear arrangement, abstractly as one-dimensional entities. Traditional models of transcriptional initiation also tend to be static, although gene transcription changes with time to adapt to developmental and environmental cues. The basis of transcriptional activity and regulation is the recruitment of transcription complexes to target genes, which is dominated by an intricate combination of transcription factors (TFs) (Levine and Tjian, 2003; Hager et al., 2009) and other regulators (Visel et al., 2009; Shen et al., 2012). In this process, the core promoter elements provide a platform for anchoring the intermediate complexes which were generally referred to as the basal transcription machinery (Dynlacht, 1997; Ptashne and Gann, 1997; Levine and Tjian, 2003; Takagi and Kornberg, 2006). Other regulatory factors could selectively bind to long-range *cis*-acting elements (such as enhancers, silencers) to regulate transcription of target gene (Dean, 2011; de Laat and Duboule, 2013). Gene activation may be achieved through DNA looping formation between enhancer-bound TFs and the transcription apparatus at the core promoter. Additionally, insulators ensure *cis*-elements to interact with the right promoters through the construction of chromosome domain boundaries (Bell et al., 2001; Bushey et al., 2008; Riethoven, 2010). Active transcription can thus be orderly achieved through the collaboration of basal and regulatory factors in RNA polymerases assembly, initiation and elongation. Besides the influences from *trans*-acting factors and *cis*-acting regulatory elements, large number of DNA/histone-modifying enzymes also contribute to local gene activity (Kimura, 2013), both of which could recruit specified protein complexes to regulate transcription without altering the underlying genes sequences. Moreover, DNA methylation and histone modification may serve as good epigenetic indicators of chromatin states, such as gene activation (H3K4me3 and H3K36me3) and repression (H3K9me3 and H3K27me3).

As discussed above, the transcriptional process seems quite simple. However, the actual status of genome in the nucleus is far from that. In eukaryotic cells, the genome is orderly organized into repeating disk-shaped units, nucleosomes, which are composed of histones, their associated DNAs, other chromatin associated proteins and RNAs (Khorasanizadeh, 2004; Woodcock, 2006). The compaction of DNAs into highly condensed chromatin obviously poses many obstacles to nuclear processes that require access to DNA sequence, including RNA transcription, DNA replication, recombination and repair. That is to say, the dense structure of chromatin limits *trans*-acting factors to access *cis*-acting regulatory elements (Edmondson and Roth, 1996; Khorasanizadeh, 2004; Berger, 2007). However, to maintain normal physical activities in cells, there also exists a dynamic balance between packaging regulatory sequences into chromatin and allowing transcriptional regulators access to these sequences (Cairns, 2009). Nevertheless, the structural nature of this inhibition and the mechanisms by which chromatin is remodeled to facilitate the regulation of gene expression have remained puzzles for many years. Extensive studies indicate that the complexity of transcriptional modulation is beyond our imagination (Metivier et al., 2006; Warnefors and Eyre-Walker, 2011). Although the genome is the same within an eukaryotic organism, their specific functions are unique due to the cells’ specific gene expression patterns. As mentioned above, the elaborate nature of genome topological organization in the nucleus makes a great contribution to the maintenance of gene transcription at the right place and the right time (Cavalli and Misteli, 2013; Gibcus and Dekker, 2013), albeit eukaryotic genomes encode genetic information in their linear sequences. Three-dimensional chromatin structure in eukaryotic nucleus makes the gene activity not solely be determined by processes occurring very close to or at the gene locus (Khorasanizadeh, 2004; Misteli, 2007). Moreover, the discovery of interchromosomal or long-range intrachromosomal interactions in higher eukaryotes points to a functional interplay between genome architecture and gene expression, challenging the view of transcription as a two-dimensional process (Gondor and Ohlsson, 2009; Schoenfelder et al., 2010).

## CHROMOSOME INTERACTIONS FUNCTION IN TRANSCRIPTIONAL REGULATION

**Figure 1:**
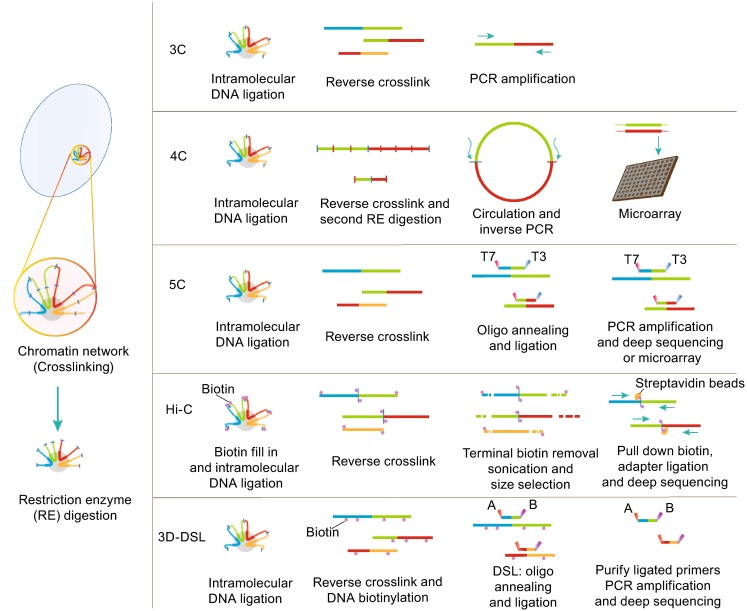
**An overview of chromosome** **conformation** **capture (3C) and related techniques**. The common steps in all 3C-related techniques are that chromosome should be crosslinked with formaldehyde and fragmented by restriction digestion. In 4C procedure, the fragment is further cleaved by a second restriction enzyme and subsequently religated to form DNA circles. The main different in 5C is the library preparation which need anneal and ligate 5C oligonucleotide after reverse crosslink. The Hi-C method adds a unique step after restriction digestion, in which the staggered DNA ends are filled in with biotinylated nucleotides (as shown by the pink dot). 3D-DSL is similar to 5C to identify chromosome interactions at pre-selected genomic locus. However, probes pools were annealed to the biotinylated 3C samples and biotinylated DNA was bound on to streptavidin magnetic beads in 3D-DSL assay. The ligated products were then eluted from streptavidin magnetic beads. This further purification step can greatly reduce background and increase signal/noise ratio

Many researches revealed that chromosomal interactions can contribute to the silencing and/or activation of genes within the three-dimensional organization of the nuclear architecture (Galande et al., 2007; Gondor and Ohlsson, 2009; Cavalli and Misteli, 2013). The genome forms extensive and dynamic physical interactions in the form of chromatin loops and bridges, which bring distal elements of the chromosome into close physical proximity, with potential consequences for gene expression and/or propagation of the genome. With advances in detection technology, it is now possible to examine these interactions at molecular level. The physical interactions of chromatin fibers can be measured by using chromosome conformation capture (3C) (Dekker et al., 2002) and related techniques including circular chromosome conformation capture (4C) (Simonis et al., 2006; Zhao et al., 2006), chromosome conformation capture carbon copy (5C) (Dostie et al., 2006), and Hi-C (Lieberman-Aiden et al., 2009) (Fig. 1). The common steps in all 3C-related techniques are that chromosomes should be crosslinked with formaldehyde and fragmented by restriction digestion (de Laat and Dekker, 2012). Classical 3C can detect chromosome interaction between two interested loci by checking the ligation products with PCR using locus-specific primers (one to one). 4C can capture genome-wide interaction profiles for a single locus with an inverse PCR. It is genome-wild but only focus on a single locus (one to many). 5C approach combines 3C and hybrid capture, so it can identify many chromosome interactions between two large loci (many to many). Hi-C is the first method that can get unbiased genome-wide chromosome interaction conformation (all to all). In Hi-C experiment, the restriction ends are filled in with biotin-labeled nucleotides before intramolecular ligation, and the ligated fragments are selected for further analysis with biotin pull-down. These genome-wide advances greatly contribute to our understanding on the mechanism of the genome organization, as well as its adaptive plasticity in response to environmental changes during development and disease. For example, analyses of *β-globin* gene loci and the regulatory regions of the *H19* using 4C and 5C have revealed large domains of interacting chromatin fibers (Dostie et al., 2006; Zhao et al., 2006). Collaborated with Dr. MG Rosenfeld and Dr. XD Fu (UCSD), we also developed three-dimensional DNA selection and ligation (3D-DSL) method (Fig. 1) and successfully detected the long-range enhancer interactional network in human chromosome 9p21 region (Harismendy et al., 2011). 3D-DSL is similar to 5C to identify chromosome interactions at pre-selected genomic locus (many to many). However, it includes further purification steps which can greatly reduce background and increase signal/noise ratio. A recent study by Fanucchi et al. indicated that co-regulated genes can form long range chromosomal contacts and that these long-range interactions may regulate these co-regulated genes’ transcription. After knocking down the factors which participate in the formation of the chromosomal contacts, the contacts will lose and these co-regulated genes transcription will not happen (Fanucchi et al., 2013).

**Figure 2:**
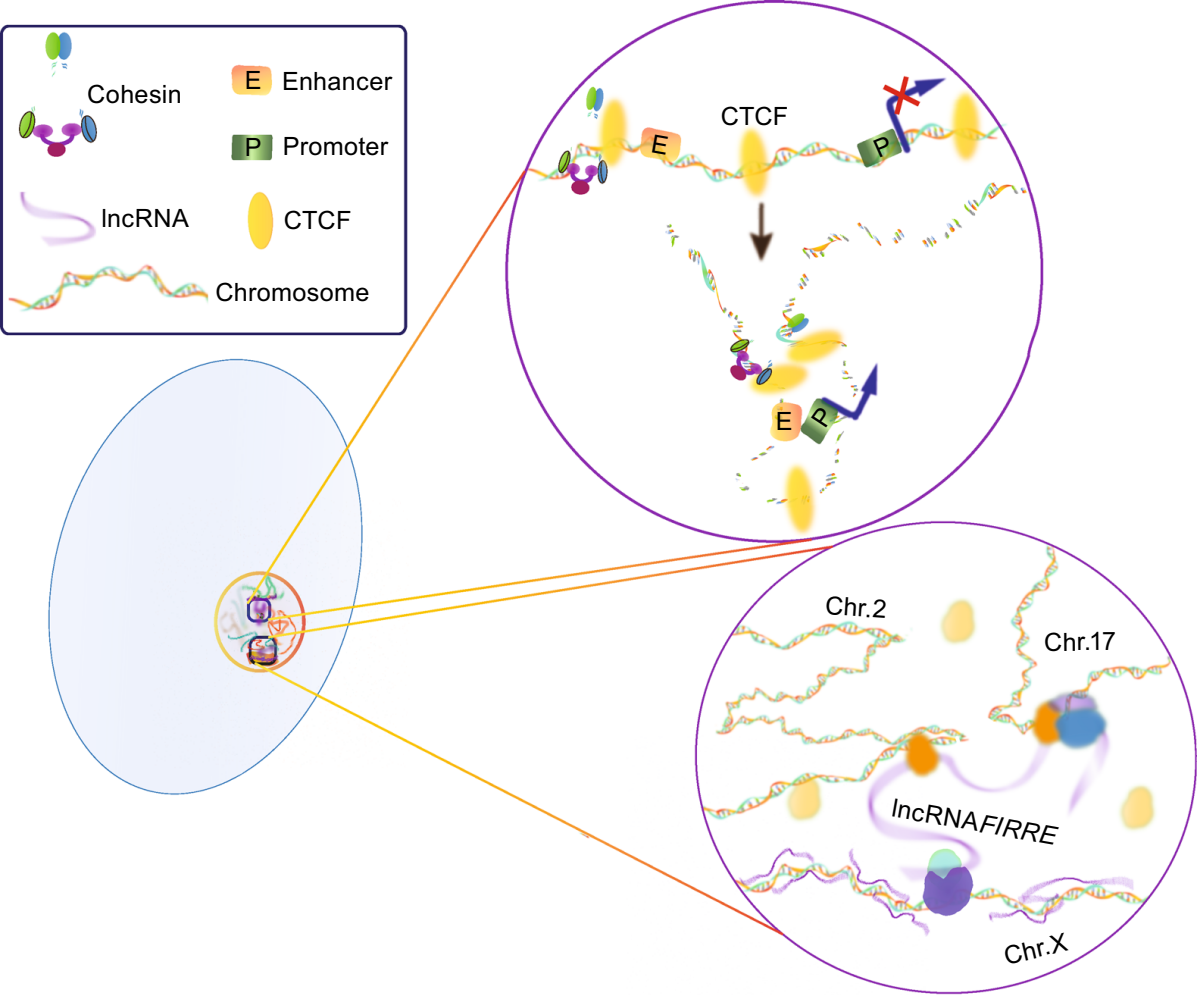
**A simplified example of how CTCF/cohesion and lncRNAs respectively participate in construction of three-dimensional chromosome network**. The lncRNA network was modified from Hacisuleyman et al. (2014)

The development of 3C-based approaches has strengthened our knowledge of the important roles of chromatin structure in transcriptional regulation. While to establish chromatin topology, architectural proteins are the keys. Architectural proteins interact with specific regulatory elements (proteins/DNA) to orchestrate long-range/short range of chromatin organization across multiple spatial scales. These interactions and chromatin topology would rearrange according to the external environment changes, and these alternations would directly affect the transcription process. The golden rule in three-dimensional regulation of transcription is that the dynamic balance between effective genome packaging and accessibility within the nuclear space should be established to adapt to environment changes and homeostasis. In this process, several crucial nodes of genome topology can be proposed (Gomez-Diaz and Corces, 2014). First and foremost, CCCTC binding factor (CTCF), which was named “Master Weaver of the Genome” (Phillips and Corces, 2009), associated with insulator sequences, boundary elements and imprinting control regions, acts as a global chromatin organizer to dominate higher-order chromatin into functional subdomains (Splinter et al., 2006; Wendt et al., 2008; Herold et al., 2012). Meanwhile, the distinct patterns of CTCF chromatin binding at dynCTS (dynamic CTCF binding sites) were positively linked with changes during gene transcription that relate to various biological processes (Nakahashi et al., 2013). Moreover, a recent research reveals that CTCF comprises a great majority of sites showing highly dynamic binding patterns during the course of cellular senescence and aging-associate diseases (Recillas-Targa et al., 2006; Thijssen et al., 2013). Additionally, mediator forms a complex with cohesion/Nipbl, which can form rings that connect the enhancer with the promoter and provides stability for long-range interactions (Fig. 2) (Parelho et al., 2008; Newman and Young, 2010; Li et al., 2013b). Particularly, 3C experiments revealed that this complex can co-occupy different promoters among different cell types and thus generate cell type-specific DNA loops and affect differential gene expression (Kagey et al., 2010). Recent studies by Tark-Dame et al. indicated that CTCF and cohesin, acting as chromatin looping proteins, are responsible primarily for constructing physical contacts, especially short-range loops, between promoters and enhancers in cell type-specific transcription (Tark-Dame et al., 2014).

Based on these evidences, it is easy to realize that *trans*-acting factors and *cis*-acting elements play vital roles in regulating nearby-gene expression and maintaining genome topology. The identification of individual basal components, especially various TFs and protein markers, makes people used to judge gene regulatory network as protein-centric, which is depending on protein-mediated transcriptional control (Millau and Gaudreau, 2011). However, as Dr. Shelley L. Berger said, “Every global traveler has experienced the disorientation of being unable to speak the local language……those in the field of chromatin are in a similar position……we are faced with observations that cannot be neatly categorized within previous models” (Berger, 2007). Thus the above-mentioned studies are just the tip of the iceberg of transcription regulation. More recent high-throughput genomic technologies have now demonstrated that only <2% of the human genome encodes for amino acids in proteins. Undeniably, alternative splicing and posttranslational modifications make a significant contribution to the diversity and functionality of the proteome. Nevertheless, at least 98% outcome of genome exists as non-protein-coding RNAs (or called non-coding RNA; ncRNAs) (Mattick, 2011). It became clear that developmental complexity and environmental adaptation probably do not solely rely on protein-mediated three-dimensional transcription. A myriad of ncRNAs may play a decisive role in most, if not all, aspects of gene regulation, especially in these epigenetic processes (Wilusz et al., 2009; Lee, 2012; Schonrock et al., 2012; Dey et al., 2014; Fatica and Bozzoni, 2014; Fitzgerald and Caffrey, 2014; Hacisuleyman et al., 2014).

## LNCRNAS FUNCTIONS IN TRANSCRIPTIONAL REGULATION

**Figure 3:**
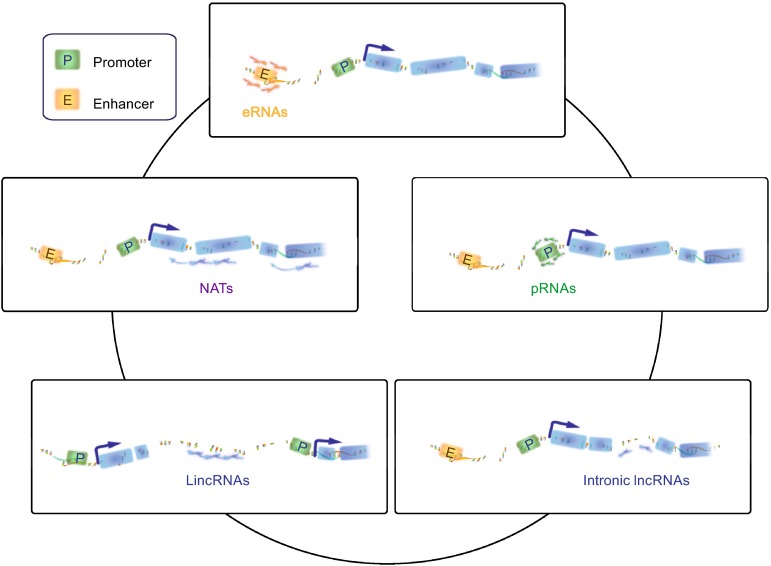
**The classification of lncRNAs**. Based on the genomic localization and context, lncRNAs can be classified as eRNAs, pRNAs, NATs, lincRNAs and intronic lncRNAs. eRNAs broadly defined as bidirectional and nonpolyadenylated transcripts which are transcribed from enhancers. pRNAs originate from intragenic promoters. NATs are transcribed from the opposite strand of either protein or non-protein coding genes. LincRNAs are transcriptional units,which are transcribed from regions intervening protein-coding loci. Intronic lncRNAs derived from specific introns of protein-coding genes

As early as 1990, Brannan and colleagues found a regulatory ncRNA when they aimed to find the mouse *H19* gene which was involved in a particular biological function by screening the cDNA library of a fetal liver. This ncRNA is different from classic structural ncRNAs such as rRNAs and tRNAs (Brannan et al., 1990). With the innovations in next-generation sequencing technologies and computational biology, a seemingly endless stream of ncRNAs are being identified and characterized at a rapid pace. Researches on ncRNAs have now gained the No.1 ranking in the top ten scientific breakthroughs in the early decades of the twenty-first century (News, 2010; Pennisi, 2010). Over the past fifteen years, small regulatory ncRNA (<200 nucleotides in length), such as small interfering RNA (siRNAs) and microRNAs (miRNAs), have been extensively investigated and the underlying molecular mechanisms have been well documented, suggesting that these ncRNAs play major roles in many cellular processes (Chitwood and Timmermans, 2010; Stuwe et al., 2014; Toscano-Garibay and Aquino-Jarquin, 2014). The surprises didn’t stop at small ncRNAs, and an expanding body of evidence reveals that long non-coding RNAs (lncRNAs, >200 nucleotides in length), once were described as ‘dark matter’, act as essential regulators in diverse cellular progresses. These include regulation of gene transcription (Orom et al., 2010; Sun et al., 2013), dosage compensation (Ilik et al., 2013; Maenner et al., 2013), genomic imprinting (Lee and Bartolomei, 2013; Simon et al., 2013), DNA damage and nuclear organization (Wang et al., 2011b; Wang et al., 2011c; Wan et al., 2013), via a number of complex yet not fully understood mechanisms. Along with the dramatic development in deep sequencing, major hurdles rise to the surface. For example, how these transcripts execute the specific function in different conditions and how to classify them (Derrien et al., 2012; Guttman and Rinn, 2012; Schonrock et al., 2012). Considering only limited information about lncRNAs’ functions and structures are known, the loci in genome where lncRNAs were transcribed become the top choice to define these transcripts. Based on the genomic localization and context, lncRNAs can be classified as enhancer RNAs (eRNAs) (Lam et al., 2014), promoter-associtated RNAs (pRNAs or PROMPTs) (Marques et al., 2013), natural antisense transcripts (NATs) (Katayama et al., 2005; Magistri et al., 2012), intergenic lncRNAs (lincRNAs) (Guttman et al., 2009; Cabili et al., 2011; Ulitsky and Bartel, 2013) and intronic lncRNAs (Guil et al., 2012). The detail classification and definition are described in Fig. 3.

Distinct from small regulatory ncRNAs that regulate gene expression mainly through base pairing to target transcripts, most identified lncRNAs play significant roles in regulating protein activity or maintaining the integrity of protein complex. LncRNAs are larger in length and thus possess complex secondary and tertiary structures. These complicated structures endow lncRNAs with the abilities to bind DNA, RNA, protein molecules and/or their combinations in the nucleus and cytoplasm, and thus they have multiple regulatory capacities (Wilusz et al., 2009; Wang and Chang, 2011; Yang et al., 2014). Here, we highlight the possible mechanisms by which lncRNAs regulate the transcription of nearby protein-coding genes. The detailed regulatory models and examples are as follows:

1. LncRNAs act as “decoy” RNAs (Fig. 4A). LncRNAs can bind to transcription factors or some other proteins away from chromatin and prevent them from binding to their proper regulatory targets. Growth arrest-specific 5 (Gas5) lncRNA, transcribed from exon 7 of *Gas5* gene, directly interacts with the DNA-binding domain (DBD) of the glucocorticoid receptor (GR) by acting as a decoy RNA version of the “glucocorticoid response element (GRE)”, thus competing with DNA GREs for binding to the GR DBD. Gas5, acting as a “riborepressor” of the GR, thus influences cell survival and metabolic activities during starvation by modulating GR-induced transcriptional activity of endogenous glucocorticoid-responsive genes (Kino et al., 2010).

**Figure 4:**
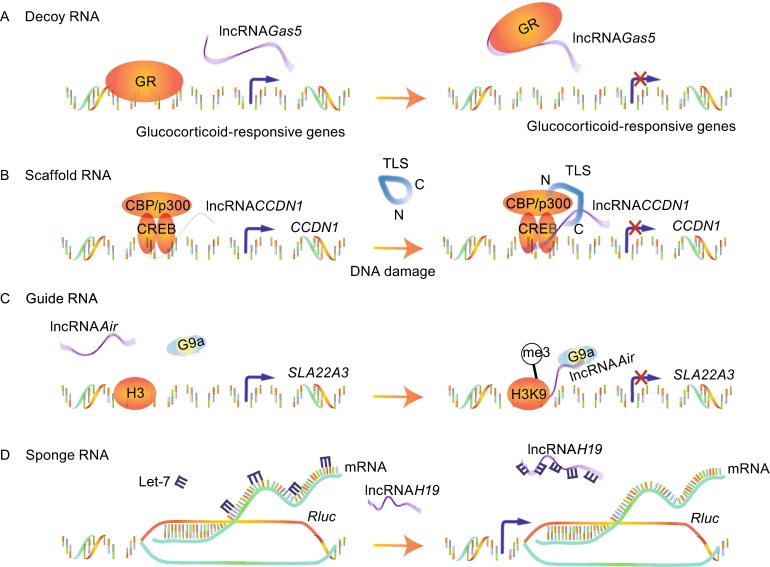
**The regulatory models of lncRNAs**
2. LncRNAs act as “scaffold” RNAs (Fig. 4B). Because of their structural flexibility, lncRNAs are well-suited to assemble diverse combinations of regulatory proteins through specific secondary structures to enhance the protein-protein interactions. A classic model is the Yeast telomerase, in which an 1157-nucleotide lncRNA (*TLC1*) not only provides the template for telomeric DNA synthesis, but also serves as a flexible scaffold for tethering TERT/Est1p/Ku/Sm into the complex (Zappulla and Cech, 2006). Our previous results provided another example, *lncRNA*_*CCND1*_. It is transcribed from the promoter region of the cyclin D (*CCND1*) gene and upregulated upon DNA damage, where it acts as molecular ‘ligand’ for RNA-binding protein TLS and promotes an allosteric effect to release it from the inactive conformation. This recruitment of TLS by *lncRNA*_*CCND1*_ leads to the inhibition of the HAT functions of CBP/p300 and repression of *CCND1* transcription (Wang et al., 2008).
3. LncRNAs act as “guide” RNAs (Fig. 4C). LncRNAs can function in *cis* on nearby gene or in *trans* on distally located genes through recruiting chromatin-modifying enzymes to targets. LncRNA *Air*, transcribed form an antisense promoter located in intron 2 of *Igf2r*, silences transcription of the distal paternal *Slc22a3* gene via a specific chromosome interaction between *Air* and the *Slc22a3* promoter (Nagano et al., 2008). Accumulated *Air* at the *Slc22a3* promoter recruits H3K9 histone methyltransferase G9a in placenta and leads to targeted H3K9 methylation and allelic silencing. Likewise, *Xist* (Wutz et al., 2002), *COLDAIR* (Heo and Sung, 2011), *HOTTIP* (Wang et al., 2011a) can guide changes of gene transcription in *cis* and *HOTAIR* (Gupta et al., 2010), lincRNA-*p21* (Huarte et al., 2010) and *Jpx* (Tian et al., 2010) can guide in *trans*.
4. LncRNAs act as “sponge” RNAs (Fig. 4D). LncRNAs could competitively inhibit the ability of miRNAs to interact with their target mRNA. The vertebrate lncRNA *H19* mentioned above harbors both canonical and noncanonical binding sites for the let-7 family of micro-RNAs, which plays important roles in development, cancer and metabolism. *H19* modulates the availability of let-7 by acting as a molecular sponge to specifically sequester endogenous let-7, preventing it from inhibiting *Rluc* expression (Kallen et al., 2013).

In addition to the models mentioned above, lncRNAs can also serve as precursors of some small ncRNAs, interact with other RNAs to form the complementary double strands, and participate in some processes such as RNA splicing and mRNA transcription (Kretz et al., 2013). Terminal differentiation-induced ncRNA (*TINCR*) interacts with a range of differentiation mRNAs (e.g. KRT80) to mediate their stabilization. *TINCR*–mRNA interaction occurs through ‘TINCR box’, a 25-nucleotide motif which is strongly enriched in interacting mRNAs and required for TINCR binding (Kretz et al., 2013). Besides that, antisense lncRNAs may form sense-antisense pairs by pairing with a protein-coding gene on the opposite strand to regulate epigenetic silencing, transcription and mRNA stability. For example, antisense *Uchl1* increases UCHL1 protein levels via an embedded inverted *SINEB2* element (Carrieri et al., 2012). In some cases, lncRNAs may have sequence-independent functions, whereby the action of their transcription alone may regulate transcription of neighboring genes (a phenomenon called transcriptional interference or promoter occlusion) (Kornienko et al., 2013).

It is worth mentioning that with the identification of circular transcript *Sry* (Capel et al., 1993), a new class of circular RNAs (circ-RNAs) has been attracting wide attentions about their biogenesis and mechanisms. Recent studies revealed that circ-RNAs not only can act as molecular sponges by competing and/or sequestering miRNAs (e.g. circular RNA sponge for miR-7, ciRS-7) (Hansen et al., 2013), but also can function as positive regulators of Pol II transcription (e.g. ci-ankrd52) (Zhang et al., 2013).

Although the functions of only a limited number of lncRNAs have been characterized to date, numerous paradigms are emerging. With the advancement of next generation sequencing and RNA profiling strategies, further work will likely identify many more lncRNAs and their functional mechanisms.

## NEW TRENDS IN TRANSCRIPTIONAL REGULATION: THREE-DIMENSIONAL VIEW

With the development on both chromosome interactions/structures and lncRNAs, we now should view and study transcriptional regulation in three-dimensional way. That is, chromosome interactions and lncRNAs together form a network of three-dimensional transcriptional regulation. Although both components are important and should be considered as a whole in transcriptional regulation in this three-dimensional view, lncRNAs play significant roles in organizing and/or maintaining the three-dimensional chromatin network formed by different chromosome interactions. Thus scientists are paying more attention to lncRNAs mediated regulation, and this is also the focus of our own lab.

Over 25 years ago, RNA was identified to be closely related with the “nuclear matrix” (Nickerson et al., 1989). Digesting or stopping the production of RNAs, but not proteins, resulted in disorganized chromatin regions inside the nucleus. Increased evidences now indicate that lncRNAs play irreplaceable roles in the establishment of three-dimensional chromatin network (Quinodoz and Guttman, 2014; Rinn and Guttman, 2014). In several dynamic systems, the transcription of lncRNAs from enhancer regions (eRNAs) has been shown to correlate with the transcription of neighboring protein-coding genes (De Santa et al., 2010; Orom et al., 2010; Wang et al., 2011c). Studies with 3C-related techniques subsequently provided evidences for a causal role in the establishment or maintenance of enhancer-promoter looping and activation of gene transcription.

A collaborated work indicated that lncRNAs in MCF-7 cells induced by 17β-oestradiol (E2) play important roles in chromosomal conformation (Li et al., 2013b). In this study, we also adopted 3D-DSL method and identified the importance of eRNAs in increasing the strength of specific enhancer-promoter looping initiated by oestrogen receptor α (ER-α). The depletion of E2-induced lncRNAs can affect the chromosomal interaction which in turn reduced the DNA looping events and gene transcription. Furthermore, Lai and colleagues identified ncRNA-activating (ncRNA-a) and revealed that the mediator complex was involved in the establishment or maintenance of chromatin looping between the lncRNA loci and their regulated promoters (Lai et al., 2013). A similar work was also provided by Xiang and colleagues shown that lncRNA *CCAT1-L* plays an important role in *MYC* transcriptional regulation and promotes long-range chromatin looping (Xiang et al., 2014). A very recent study provided new insights into the formation of “chromatin loops” mediated by lncRNAs (Hacisuleyman et al., 2014), where the control range of lncRNAs is no longer restricted to single chromosome. It is found in this work that lncRNA *Firre* interacts with hnRNPU (nuclear-matrix factor), and brings at least three genes together around their own transcription sites. The co-localization of these different gene loci is lost upon deleting the lncRNA *Firre* locus or knocking down hnRNPU. This multichromosomal nuclear interaction could be achieved by RNA-protein-DNA loop and bring the specific gene locus into proximity that may be far away in linear sequence (Fig. 2).

**Figure 5:**
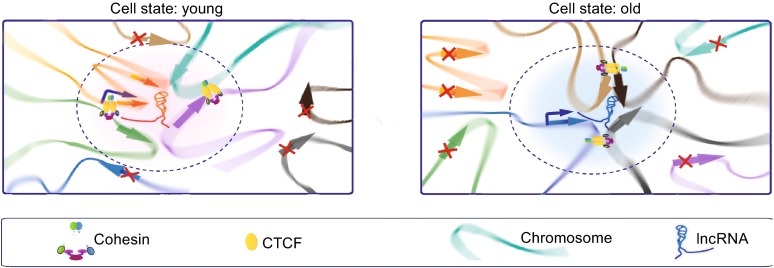
**The hypothetical model of how CTCF/cohesion collaborate with different lncRNAs to establish relatively stable state of three-dimensional chromatin network and regulate gene expression in the process of cell aging**

Meanwhile, a bulk of evidences suggest that the characteristics of lncRNAs include relatively low abundance, tissue-restricted or cell type-specific expression patterns and localization to specific subcellular compartments. These intrinsic indices of lncRNAs suggest that they are likely representing a previously hidden epigenetic regulator in the determination of cell development, function and adaptation. Given that the degree of organismal complexity scales with the amount of non-coding DNA sequences, it is intriguing to speculate that the increase in regulatory complexity afforded by the dynamic interplay between lncRNAs and chromosome interactions, which established the dynamic three-dimensional transcriptional regulation, may be responsible for the more complex transcriptional regulation and epigenetic changes in organisms with higher complexity. However, even with the massive increasing information on lncRNAs, we still have a lot to learn. For example, to what degree do lncRNAs participate in construction of genome topology and gene expression? What do they depend on when they form clusters to target gene in spatial proximity? In the process of recruitment to specific genome loci, which way do they choose to execute—working collaboratively or alone? If they choose to collaborate, who will dominate this enrichment process? A recent study demonstrated that a large number of lncRNAs were identified during replicative senescence, with few functions fully understood (Abdelmohsen et al., 2013). With the acceleration of population aging process, the relationship between lncRNAs and senescence-associated diseases has attracted more and more attention (Abdelmohsen et al., 2013). Our lab aims to use 3D-DSL technology together with other genomic techniques to study the molecular mechanism of transcription during cellular senescence, focusing on the roles of lnRNAs, and their interactions with regulatory proteins (CTCF/Cohesin) and genome loci that form genome topology in this complex transcriptional regulation. We are particularly interested in finding out whether lncRNAs participate in the chromatin network with CTCF. We speculate that during the cell aging, lncRNAs drag different regions of inter/intra-chromosome to assemble specific transcriptional active and repressive regions, relying on the flexible structure of lncRNAs. In this process, CTCF collaborates with lncRNAs to establish relatively stable state of three-dimensional chromatin network and regulate gene expression (Fig. 5). This study will hopefully provide new clues on how aging occurs.

## FUTURE DIRECTIONS

Eukaryotic gene transcription can be viewed within a conceptual framework in which regulatory mechanisms are integrated at three hierarchical levels. The first is the sequence level, i.e. the linear organization of transcription units and regulatory sequences. The second is the chromatin level, which allows switching between different functional states. This regulatory level is close related to histone modification, DNA methylation, lncRNAs and a variety of repressing and activating mechanisms. The third level is the nuclear level, which includes the dynamic three-dimensional spatial organization of the genome inside the nucleus. The nucleus is structurally and functionally compartmentalized, and epigenetic regulation of gene expression may involve repositioning of loci in the nucleus through changes in large-scale chromatin structure. The traditional theory of two-dimensional transcriptional regulation has been undergoing a fundamental shift to three-dimensional modulation. Our understanding of the roles and implications of dynamic properties of the transcription machinery is still in infancy. Thus, it is more necessary to integrate the different epigenetic determinants, especially chromatin conformation characteristics and lncRNAs into a whole to explicate the mechanism of gene transcription.

Emerging developments in technologies have made it possible to create high resolution genome-wide maps of physical interactions along genomic regions and ncRNAs. First of all, in the aspect of three-dimensional chromatin network research, advanced imaging technologies (for example single-molecule RNA fluorescence *in situ* hybridization; single-molecule RNA-FISH) could examine subcellular localization of lncRNA *Firre* in embryonic stem cells (Hacisuleyman et al., 2014). Meanwhile, combination of technology innovations in probing the folding of chromosomes (such as 3D-DSL and Hi-C) has uncovered an extensive, and previously underestimated network of local and long-range intrachromosomal loops and interchromosomal contacts. Howbeit, light microscopy just affords a resolution of 100–200 nm at best, which is insufficient to define clear chromosome conformation. Electron microscopy, while affording high resolution, is laborious and not easily applicable to study specific loci, not to mention the definite targets of lncRNAs. DNA binding proteins fused to different versions of green fluorescent proteins permit visualization of individual loci, but only a few positions can be examined simultaneously. Multiple loci can be visualized with FISH, but the resolution for different loci is about 1000 kb. Given the limitation of above techniques and based on the physical crosslinking in the close region of inter/intra-chromosome, the series of high-throughput 3C-associated methodologies can be used to analyze the overall spatial organization of chromosomes and to investigate their physical properties at high resolution in a more systematic and unbiased manner. Notwithstanding, a common problem in all of these techniques is the requirement of a great number of cells, especially in the high-throughput methodologies. Single cell Hi-C method has thus been developed (Nagano et al., 2013), which effectively bridge current gaps between genomics and microscopy studies of chromosomes. Secondly, in the study of lncRNAs function, RNA microarray plays a prominent role in providing reliable and sensitive results to discover the mechanism studies of lncRNAs. With accurate probe annotations and designs to distinguish splicing variants and detect ncRNAs, microarray analysis can get pretty close to what RNA-Seq or lncRNAs-Seq can offer at a significantly lower cost. The mature software and normalization techniques make analyzing RNA microarray data seem like a breeze. However, microarrays only indicate known and relative rather than absolute transcript levels. If we want to assess rare and whole transcripts, GRO-Seq and lncRNA-Seq are better options: the dynamic range is orders of magnitude greater than that of microarrays. We believe that the cost and complication of analysis can improve over time, and with the advent of third generation sequencing, which has a lower rate of sequencing error (not enzyme based) and no need for amplification, the future of GRO-Seq and lncRNA-Seq will certainly be promising.

Comprehensive understanding of three hierarchical levels of transcriptional regulator is contingent on the upgrading and innovation of technologies. These will come in several areas ranging from classic molecular biology techniques to bioinformatics tools. Despite that many mysteries about gene transcription are still under investigation, the overall picture is gradually clear: interdisciplinary techniques that enable imaging analysis of complex *in vivo* systems will help bridge the gap between live cell imaging and *in vitro* biochemistry. With research underway in all of these areas, the rapid progress in this field over the past decade should continue unabated.

## ABBREVIATIONS

CTCF: CCCTC binding factor
GR: glucocorticoid receptor
GRE: glucocorticoid response element
lncRNAs: long non-coding RNAs
miRNAs: microRNAs
ncRNAs: non-coding RNAs
siRNAs: small interfering RNA
TFs: transcription factors
